# Survey data on bureaucratic processes and entrepreneurial venture performance in SMEs in Nigeria

**DOI:** 10.1016/j.dib.2018.08.038

**Published:** 2018-08-22

**Authors:** Fatai Alani Lawal, Oluwole O. Iyiola, Omotayo A. Adegbuyi, Oladele J. Kehinde, Olaleke O. Ogunnaike

**Affiliations:** Department of Business Management, Covenant University, Ota, Ogun State, Nigeria

**Keywords:** Bureaucratic processes, Entrepreneurial ventures, SMEs, Venture performance

## Abstract

This article presents data that examined the impact of bureaucratic processes on the performance of small and medium enterprises in Nigeria. A survey was carried out by administering the questionnaire to 400 business owners selected from three reputable associations, namely: NASME, NASSI and ASBON (Nigerian Association of Small and Medium Enterprises, National Association of Small Scale Industrialists and Association of Small Business Owners of Nigeria) in three geo-political zones. Data were analysed using Predictive Analytics Software. Correlation and Regression were employed as inferential statistical tool of analysis. Data set revealed the existence of relationship and the extent to which bureaucratic processes impact the performance of entrepreneurial ventures.

**Specification Table**

**Table:** 

**Subject area**	Business, Management
**Major Specific Subject Area**	Business and Entrepreneurship
**Type of Data**	Table
**How Data was acquired**	Researcher made questionnaire analysis
**Data format**	Raw, analysed, descriptive and inferential statistical data.
**Experimental factors**	Survey method involved the use of questionnaire to gather data from 400 SMEs owner-managers that affiliated with three professional associations (selected based on their geographical spread) in three geo-political zones in Nigeria.
**Experimental features**	The researcher-made questionnaire which contained data on bureaucratic process and performance measures were completed.
**Data Source location**	South-West, North-Central and South-South Nigeria
**Data Accessibility**	Data is included in this article.

**Value of data**

• These data are a presentation of descriptive data on bureaucratic processes that characterises the formal institutional environment of the local entrepreneurial climate as it relates to the performance of entrepreneurial ventures.
• The data showed that understanding of the peculiarities of the bureaucratic dimensions (occasioned by compliance and regulatory procedures) and the adoption of appropriate coping strategies by SMEs owner-managers can be helpful for business performance improvement.
• The dataset of this study can be used to institute necessary institutional policy framework that promotes the ease of doing business by start-ups and growing SMEs.

## 1 Data

Table 1 shows the correlation relationship that exist between bureaucratic processes and venture performance amongst SMEs in the regions. Measures of bureaucratic process dimensions include rules and procedures (Rul_Proc), cost of business registration (Reg_Cost), and business documentation and renewal process (Biz_Doc), while venture performance (Vent_Perf) measurement was based on growth, profitability and competitiveness parameters. Statistical analysis reveals the existence of relationship (though insignificant) between bureaucratic processes and venture performance amongst SMEs in the three regions as depicted by correlation coefficient *r* = 0.046; *p* < 0.01 (South-West), *r* = −.011; *p* < 0.05 (North-Central) and *r* = −.059; *p* < 0.01 (South-South).

**Table 1:** Data on correlational analysis between bureaucratic process and venture performance.

**Regions**	***r***	**sig. value**	***p***	**Remark**
South-West	0.046	0.446	*p* < 0.01	Insignificant
North-Central	−0.011	0.929	*p* < 0.05	Insignificant
South-South	−0.059	0.752	*p* < 0.01	Insignificant

The hypothesis formulated in the null form states that “there is no significant effect of bureaucratic processes on venture performance’’ and was tested using regression analysis.

Table 2 represents the Model Summary and it describes the extent to which variance in the dependent variable (venture performance) is explained by the independent variable (bureaucratic processes). Thus *R*^2^ = .008 (i.e. 0%) and adjusted *R*^2^ = .001 (i.e. 0%) implying that there is no variation in venture performance attributable to the dimensions of bureaucratic process. The findings from this study is consistent with the assertions of existing studies [1], [2], [4].

**Table 2:** Model Summary.

***R***	***R***^**2**^	**Adjusted *R***^**2**^	**Standard error of estimate**
0.092^a^	0.008	0.001	0.682662

a Predictors: (Constant), Biz_Doc, Rul_Proc, Reg_Cost

Table 3 shows the analysis of variance (ANOVA) test which indicates that *F* value = 1.070 @ *p*-value = 0.362 level of significance > 0.05. The result of the tested hypothesis revealed that bureaucratic processes have no significant effect on the performance on entrepreneurial ventures.

**Table 3:** ANOVA for the relationship between bureaucratic processes and venture performance.

	**Sum of squares**	**Df**	**Mean square**	***F***	**Sig.**
Regression	1.496	3	0.499	1.070	0.362^a^
Residual	175.692	377	0.466		
Total	177.188	380			

a Predictors: (Constant): Biz_Doc, Rul_Proc, Reg_Cost

The coefficient Table 4 depicts the simple model that expresses the extent to which bureaucratic processes have effect on venture performance and which of the variables included in the model contributed to the prediction of the dependent variable using beta values for the comparison. The model revealed that all *T*_values_ do not have statistical significance as they are < 1.96 @ significance levels > 0.05 representing statistical confidence < 95%., whereas the significance level < 0.05 implies a statistical confidence of above 95%. This implies that dimensions of bureaucratic processes make no unique contributions towards explaining variance / change in venture performance.

**Table 4:** Data of coefficient of determination of bureaucratic processes and venture performance.

**Model**	**Unstandardized coeff.**		**Standardized coeff.**	**t**	**Sig.**
	**β**	**Std. Error**	**β**		
(Constant)	3.236	0.166		19.524	0.000
Biz_Doc	0.046	0.036	0.073	1.283	0.200
Rul_Proc	−0.052	0.037	−0.081	−1.407	0.160
Reg_Cost	0.024	0.034	0.040	0.719	0.473

## 2 Experimental design, materials and methods

Survey method involved the use of questionnaire to gather data from 400 SMEs owner-managers that affiliated with three professional associations (selected based on their geographical spread) in three geo-political zones in Nigeria [3]. Convenience and proportionate sampling techniques were used in selecting respondents. Convenience sampling to accommodate members that are consistent in paying membership dues and regularity at monthly association meetings while proportionate sampling based on the membership register of each association [5].

The professional associations serve as a forum to assess the SMEs owner-managers, thus making the survey method more efficient in securing opinions, description, attitudes as well as annexing cause and effect relationship for quantitative comparison [6]. This research benefitted from the ideas of existing research studies particularly in designing the research instrument [7], [8], [9] using 5 point likert scale which ranges from strongly agree - 5, agree - 4, undecided - 3, disagree - 2, and strongly disagree - 1. Data obtained were coded and analysed by Predictive Analytic Software (Statistical Package for Social Sciences-SPSS v 22) using inferential statistics (involving correlation and regression) to test formulated hypothesis.
