# Supplementary material for: Survey data on bureaucratic processes and entrepreneurial venture performance in SMEs in Nigeria
Source: Data Brief. 2018 Aug 22;20:775–8. doi: 10.1016/j.dib.2018.08.038 (PMC6129735; doi:10.1016/j.dib.2018.08.038)
Supplement: Supplementary file 2 — Supplementary material [file mmc2.docx]

**Research Survey Questionnaire**

**SECTION A: Demographic Information**

**Instruction**: The following are for classification purpose only. Please tick (√) as appropriate.

1**.** Gender (a) Male ( ) (b) Female ( )

2. Age

(a) Below 30 ( ) (b) 31 – 40 ( ) (c) 41 – 50 ( ) (d) 51 and above ( )

3. Marital Status (a) Single ( ) (b) Married ( ) (c) Divorced ( )

(d) Others, please specify………………………...

4. Educational Level:

(a ) Secondary ( ) (b ) Post-secondary ( ) ( c) Fist Degree / HND ( )

(d) Postgraduate ( ) (e ) Others, please specify…………….………………..

5. How many years of working experience did you have before starting this business?

(a) less than 5 years ( ) (b) 5- 10 years ( ) (c ) 10 – 15 years ( )

(d) 15 -20 years ( ) (e) above 20 years ( ) (f ) no working experience ( ).

6. How long has your company been in business?

(a) less than 5 years ( ) (b) 5 – 10years ( ) (c ) 10 -15 years ( ) (d) Above 15 years ( )

7. How many employees work in your company?

(a) less than 10 ( ) (b) 10 - 49 ( ) (c ) 50 – 199 ( ) (e ) Above 200 ( )

8. What is the main activity of your company?

(a) Manufacturing ( ) (b) Services ( ) (c) Trading ( ) (d ) Agriculture ( )

(e ) other services, please specify………………………………….

9. What is the Legal status of your business?

(a) Sole Proprietorship ( ) (b) Limited Liability ( ) (c ) Public Liability ( )

(d) Un-registered ( ) (e ) Others, please specify…………………………………

**SECTION B**

**Instruction:** Kindly tick (√) as appropriate whether you “Strongly Agree”(SA), Agree (A) “Undecided” (U), ‘’Disagree’’ (D), or ‘’Strongly Disagree’’ (SD).

|  | **Bureaucratic Processes** | **SA** | **A** | **U** | **D** | **SD** |
| --- | --- | --- | --- | --- | --- | --- |
| **1** | There are delays in business registration, document procurement and renewal processes. |  |  |  |  |  |
| **2** | There are bottlenecks and protocols in getting things done within government establishments / parastatals |  |  |  |  |  |
| **3** | The cost of registering a business is often high for SMEs to meet. |  |  |  |  |  |
| **4** | There is high tax burden and multiple taxation on business activities. |  |  |  |  |  |
|  |  |  |  |  |  |  |
|  | **Venture Performance** | **SA** | **A** | **U** | **D** | **SD** |
| **5** | I am satisfied with my firms’ performance for the past three years in comparison to her competitors |  |  |  |  |  |
| **6** | I reached the expected profitability target |  |  |  |  |  |
| **7** | I reached higher profitability than others in my business sector in the last three years |  |  |  |  |  |
| **8** | Profitability has increased in the last three years |  |  |  |  |  |
| **9** | Total sales volume has increased in the last three years |  |  |  |  |  |
| **10** | Employees number has increased in the last three years |  |  |  |  |  |
| **11** | Our market share has increased in the last 3 years |  |  |  |  |  |
| **12** | Our customers base has grown significantly in the last 3 years |  |  |  |  |  |
| **13** | In dealing with our competitors, we typically initiate actions, which competitors then responded to. |  |  |  |  |  |
| **14** | In dealing with our competitors, we are very often the first to introduce new products / services |  |  |  |  |  |
| **15** | The company knows the main competitors, and is aware of its own competitive position in the market. |  |  |  |  |  |
| **16** | The company gathers competitors information continuously |  |  |  |  |  |
